# Impact of the COVID-19 Pandemic on Gut Cancer Admissions and Management: A Comparative Study of Two Pandemic Years to a Similar Pre-Pandemic Period

**DOI:** 10.3390/healthcare13070805

**Published:** 2025-04-03

**Authors:** Sergiu Marian Cazacu, Ion Rogoveanu, Adina Turcu-Stiolica, Alexandru Marian Vieru, Anca Gabroveanu, Petrică Popa, Mircea Pirscoveanu, Dan Cartu, Liliana Streba

**Affiliations:** 1 Gastroenterology Department, University of Medicine and Pharmacy Craiova, Petru Rares Street No 2-4, 200349 Craiova, Romania; sergiu.cazacu@umfcv.ro (S.M.C.); ion.rogoveanu@umfcv.ro (I.R.); petrica.popa@umfcv.ro (P.P.); 2 Biostatistics Department, University of Medicine and Pharmacy Craiova, Petru Rares Street No 2-4, 200349 Craiova, Romania; 3 Doctoral School, University of Medicine and Pharmacy Craiova, Petru Rares Street No 2-4, 200349 Craiova, Romania; 4 Resident Physician, Emergency County Clinic Hospital Craiova, 200349 Craiova, Romania; dr.ancagabroveanu@yahoo.com; 5 Surgery Department, University of Medicine and Pharmacy Craiova, Petru Rares Street No 2-4, 200349 Craiova, Romania; mircea.pirscoveanu@umfcv.ro (M.P.); cartu_dan@hotmail.com (D.C.); 6 Oncology Department, University of Medicine and Pharmacy Craiova, Petru Rares Street No 2-4, 200349 Craiova, Romania; liliana.streba@umfcv.ro

**Keywords:** COVID-19, esophageal tumors, gastric tumors, colorectal tumors, surgery

## Abstract

**Background/Objective**: Gastrointestinal tract cancers may have been severely affected by the COVID-19 pandemic. The limitations of digestive endoscopy, the fear effect, and restrictions on hospital admissions during the pandemic may have delayed the presentation of patients to hospitals and surgical procedures and may have impacted overall survival. **Methods**: We conducted an observational, cross-sectional study of esophageal, gastric, small bowel, and colorectal cancer patients admitted to our hospital between 1 January 2018 and 31 December 2021. We analyzed the hospitalization rates, pathological type, the onset by complications, staging, and surgery during the pandemic compared to a pre-pandemic period (January 2018–December 2019). **Results**: During 2018–2021, 1613 patients with malignant gut tumors were admitted to our hospital (112 esophageal and eso-cardial tumors, 419 gastric tumors, 34 small bowel tumors, and 1058 colorectal tumors). Admission was reduced by 30.3% for esophageal and eso-cardial malignant tumors, 27.6% for gastric tumors, and 17.3% for malignant colorectal tumors. For esophageal and eso-cardial tumors, a higher frequency of stenosing tumors and palliative gastrostomies was noted. More stage III gastric cancers and a lower rate of vascular invasion were recorded during the pandemic. No differences regarding small bowel tumors were noted. In colorectal tumors, slightly more stage II cancers and more stenosing tumors were recorded, but occlusive, bleeding, and perforated tumors were similar; also, surgical rates were similar, with a two-fold higher perioperative mortality. The overall survival of gastric and colorectal carcinoma was higher during the pandemic (but with no statistical significance), although a clear explanation has not emerged. **Conclusions**: The impact of the COVID-19 pandemic on gut cancer included a significantly lower rate of newly diagnosed admissions, more stage II colorectal and stage III gastric carcinomas, a two-fold higher perioperative mortality for colorectal carcinoma, and a trend for a surprisingly higher overall survival for gastric and colorectal tumors (but without statistical significance). Future research is necessary for assessing long-term impact.

## 1. Introduction

The COVID-19 infection appeared in late 2019 and was declared a global pandemic by the World Health Organisation (WHO) on 11 March 2020, with more than 7 million deaths by August 2023 [1]. The severity of cases has decreased significantly since the appearance of the omicron variant of the virus [2]; by 5 May 2023, the WHO announced that the infection is no longer a public health emergency of international concern [3]. The pandemic had a severe impact on global healthcare management because of lockdown measures; the reprioritization of healthcare services; the need for dedicated beds, personnel, and circuits for SARS-CoV-2 patients; inappropriate and insufficient capabilities in emergent cases; and the effect of patients’ fears surrounding hospital presentations and admissions [3], which delayed or limited medical care for patients with non-COVID-19 pathologies, including gastroenterological emergencies and cancer diagnosis and management [3,4,5,6].

The impact of the COVID-19 pandemic on cancer care was severe, with significant delays or cancellations in screening, diagnosis, and treatment [4] and a 20–30% reduction in newly diagnosed cancer reported by the European Network of Cancer Registries during the pandemic [5]. However, there is marked heterogeneity in studies assessing the impact of the COVID-19 pandemic on cancer management because of the cancer site, reporting period of the pandemic, cancer care programs, or specific restrictions related to each country; an umbrella review of the systematic reviews found that 16 reviews ended searches during 2020, 25 ended during 2021, and 5 ended during 2022 [4], which could alter the data. In Europe, a worse process and outcome measures were noted for all cancers except for lung cancer (related to the extensive CT scanning used for the COVID-19 pneumonia diagnosis), with no relation to the lower healthcare expenditures or lower investments in prevention but with fewer delays in countries with more than 20% general practitioners [5].

Gastrointestinal tract cancers may have been severely affected by the COVID-19 pandemic. The limitations of digestive endoscopy at the beginning of the pandemic because of aerosol-inducing procedure restrictions may have affected both diagnostic and screening endoscopic procedures [7,8,9,10,11], but many endoscopic-based screening programs were resumed later during the pandemic. The fear effect, combined with restrictions on hospital admissions during the pandemic, may have delayed the presentation of patients to hospitals and surgical procedures and shifted both the diagnosis and oncological treatment to an outpatient setting instead of hospitalization [12], which could impact the admissions for gastrointestinal cancers. A reduction of early tumors at more advanced stages was noted in most studies [4,5,7,13,14,15,16,17,18,19,20,21,22,23,24,25,26,27,28], although others did not see such an effect [10,29,30,31,32,33,34,35]. A higher proportion of emergency presentations was also noted in some studies [19,25,27,36], but not in others [37]. Regarding surgical management, some studies found no changes during the pandemic [14,23,35], whereas other studies have shown a decreased percentage of radical procedures [9,33]. A return to the pre-pandemic staging data was also observed in some studies [14,34]. Similar data were provided for pancreatic carcinoma, with more advanced staging (69.8 versus 59.7% in France) [38] and more stage IV disease (53.8 versus 8.3% in a small unicentric study) [39], and also for hepatocellular carcinoma (with larger size and more BCLC stage B in the pandemic period) [40], although the impact may be less severe for hepatocellular carcinoma because of easier access to transabdominal ultrasound and CT scan examination [40,41,42,43]. No data regarding small bowel tumors are available, although the potential impact of the reduction in upper and lower digestive endoscopy may be mitigated by the rarity of these types of tumors and by diagnosis via CT scan and videocapsule endoscopy [44,45].

In Romania, six published studies have assessed the impact of the COVID-19 pandemic on cancer management. Four studies analyzed colorectal carcinoma (CRC) surgical management during the COVID-19 pandemic [16,46,47,48], and the other two studies were related to melanoma management during the pandemic [49,50]. No studies assessing the management of esophageal or gastric carcinoma during the pandemic in Romania are currently available.

The purpose of our study was to assess the impact of the COVID-19 pandemic on admission, imaging examination, and staging for esophageal, gastric, and colorectal cancers admitted during March 2020–December 2021 as compared to a similar pre-pandemic period (March 2018–December 2019) in a tertiary care hospital in Romania. The effect of the pandemic on the diagnosis of eso-gastrointestinal tumors is very uneven in different countries because of the marked differences regarding cancer diagnosis (screening or symptom-based), therapeutic protocols (surgery, chemotherapy), healthcare changes during the pandemic (lockdown, patient restrictions, resources availability, outpatients and inpatient settings), and also differences in patient psychology regarding presentation to hospitals (fear effect) during the pandemic and after. For these reasons, a study in a tertiary care hospital in Romania may add to our understanding of the effects of the pandemic period and restrictions on the diagnosis and treatment of patients with gut tumors in our geographic area.

## 2. Materials and Methods

### 2.1. Study Design

We conducted an observational, cross-sectional study that included all esophageal, gastric, and colorectal cancer patients admitted to the Craiova Emergency County Clinic Hospital between 1 January 2018 and 31 December 2021. We analyzed the hospitalization rates for esophageal, gastric, small bowel, and colorectal cancers; histopathological type; onset by complications; and staging during the pandemic compared to pre-pandemic patients, CT aspects, and surgical management. Our analysis included a pandemic period between January 2020 and December 2021 as opposed to a similar pre-pandemic period between January 2018 and February 2020. This study was performed in accordance with STROBE guidelines for observational studies [51].

### 2.2. Patient Selection and Data Collection

The data were collected from the analysis of the patient’s discharge documents from the Hippocrates computer system (Version 4, Romanian Software Solutions, Bucharest, Romania) of the hospital (diagnostic codes: C15—malignant esophageal tumor, D19—benign esophageal tumor, C16—malignant stomach tumor, C18–C20—malignant colorectal tumor and D37—digestive tumors with unpredictable and unknown evolution) and were supplemented as needed by analyzing the patient’s medical records. Exclusion criteria were as follows: patients under 16 years of age, those with no pathological confirmation of the malignancy, and those with insufficient data. Eso-cardial tumors were defined as tumors with an epicenter not more than 2 cm proximal and distal to the eso-cardial junction. All patient data were collected in an Excel table, including demographic, clinical, imaging, and laboratory information; in the case of surgical procedures, postoperative staging and pathological information were also registered. The staging of esophageal, gastric, and colorectal tumors was based on the eighth AJCC/TNM staging for clinical staging (Supplementary Tables S1–S6) [52,53,54,55]. Data were completed with information obtained from the Romanian National Database Cancer.

We defined overall survival (OS) as the length of time from the date of diagnosis of the disease (colorectal or gastric cancer) that patients were still alive.

### 2.3. Statistical Analysis

The extracted data were saved and computed in an Excel spreadsheet. Data were expressed as mean ± standard deviation (SD), range (minim–maxim) for continuous variables, and percentages for discrete variables. We evaluated the *p*-value for the staging, surgical management, and admissions during the pandemic compared to a similar pre-pandemic period. We used R packages (R Core Team 2022, v. 4.2.2 for Windows) for the statistical analysis [56,57]. The Kaplan–Meier method was used to estimate OS with 95% confidence intervals (95%CI). The log-rank test was used to compare OS among the two periods of time: pre-pandemic and pandemic. We performed univariate and multivariate analysis to estimate the hazard ratio (HR), with a corresponding 95%CI for assessing the influence of factors such as age, gender, staging, T stage, N stage, M stage, surgery, occlusion, and perforation on OS. A *p*-value below 0.05 was considered statistically significant.

## 3. Results

### 3.1. Main Characteristics of the Patients

**Figure 1:**
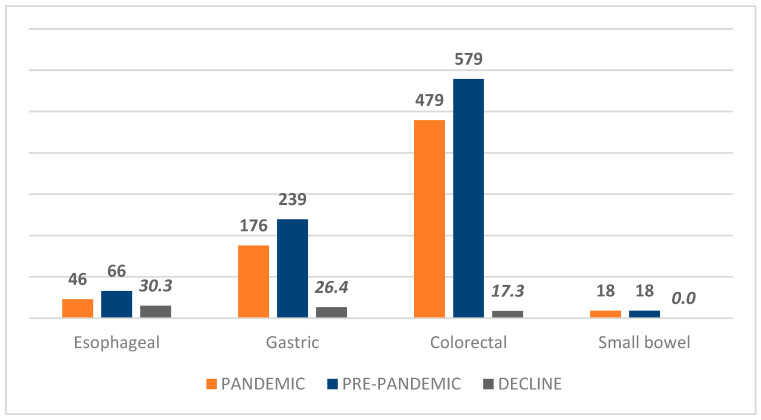
The number of cases of gut cancers and the difference (%) between the pre-pandemic and pandemic cases.

A total of 1613 patients with malignant gut tumors were admitted to our hospital during 2018–2021 (112 esophageal and eso-cardial tumors, 419 gastric, 34 small bowel, and 1058 colorectal tumors). Admission was reduced by 30.3% for esophageal and eso-cardial malignant tumors, by 27.6% for gastric tumors, and by 17.3% for malignant colorectal tumors (Table 1, Table 2 and Table 3, Figure 1). In contrast, the same number of small bowel malignant tumors was noted (17 tumors for each period).

For esophageal and eso-cardial tumors, the mean age was similar, with more men affected during the pre-pandemic period (90.9 versus 76.1%, *p* = 0.0374). Most tumors were carcinomas, with a similar proportion between squamous carcinoma and adenocarcinoma (approximately four to one). No significant differences were noted regarding grading and staging (T-staging, N-staging, N-staging, and the proportion of I–IV stages). CT scans detected a slightly higher percentage of esophageal and eso-cardial tumors during the pandemic period (91.7 versus 76.2%, *p* = 0.0792). The location was similar, but more type II Borrmann cancers were noted during the pre-pandemic period. A higher frequency of stenosing tumors (76.1 versus 56.1%, *p* = 0.0317) and palliative gastrostomies (39.1 versus 18.2%, *p* = 0.0156) was also recorded (Table 1). Radical surgery was only rarely performed, which suggests advanced diseases in both periods.

For gastric malignant tumors, age and gender were similar. Most tumors were adenocarcinomas, with similar proportions, more G1 tumors during the pandemic and more G2 tumors during the pre-pandemic period; 15 lymphomas, 4 neuroendocrine tumors, 23 malignant GISTs, and 2 sarcomas were recorded. More stage III tumors were recorded during the pandemic (40.3 versus 31.2%, *p* = 0.0300), although the differences between the T1–4, N1–3, and M stages were not statistically significant. A lower rate of vascular invasion was recorded during the pandemic (38.2 versus 63%, *p* = 0.0138). The location, the rate of complications, and the surgery were similar. The detection rate of gastric tumors by CT scan was also similar (56.9 and 69.1%, respectively). Perioperative mortality was similar (12.5 versus 10.7%, *p* = 0.5358). Emergency surgery rates and the percentage of invaded margins were similar (Table 2).

During 2018–2021, 34 small bowel tumors were diagnosed and pathologically confirmed (17 during 2018–2019 and 17 during 2020–2021). The mean ages were 63.8 ± 9.2 and 67.4 ± 11.2. (*p* = 0.3131). There were five duodenal tumors, six jejunal tumors, five ileal tumors, and one case with multiple-location tumors in the pre-pandemic period, while in the pandemic period, we noted four duodenal tumors, four jejunal tumors, one jejuno-ileal tumor, seven ileal tumors, and one multiple-location tumor. We also recorded one bleeding tumors, three occlusions, one invagination, and five obstructive tumors during 2020–2021, and two bleeding tumors, three occlusions, and three obstructive tumors during 2018–2019. Eight carcinomas were diagnosed during 2020–2021 and 11 were diagnosed during 2017–2018. Three malignant GISTs were diagnosed during the pandemic, and none were recorded during 2017–2018, although we noted three and one benign GIST diagnosed during the pre-pandemic and pandemic, respectively. One lymphoma, two metastases, and three sarcomas were diagnosed during the pre-pandemic period, while in the pandemic period, three lymphoma, one metastasis, two neuroendocrine tumors, and one sarcoma were discovered and operated on. Four tumors had lymphatic (2/4), vascular (1/4), and perineural invasion (2/4) assessed in the pandemic period, and we assessed four during the pre-pandemic (1/4 lymphatic, 4/4 vascular, and 3/4 perineural invasion). Five tumors were G2 and two were G3 during the pre-pandemic period, while five tumors were G2 and three were G3 during the pandemic period. In the pre-pandemic period, surgical resection was performed in nine cases (52.9%) and internal derivation was performed in three other cases, while three cases were assessed as inoperable because of distant metastasis and local extension and two patients did not consent to surgery. During the pandemic period, 13 surgical resections for small bowel tumors were noted (76.5%, *p* = 0.1574), with two cases having distant metastases and two patients not consenting to surgery. During the pandemic period, there were two T4 tumors, one T3–T4, and four T3 tumors; three had N0 tumors, three had N+ tumors, and three from seven cases had distant metastasis. During the pre-pandemic period, one T4 tumor and three T3 tumors were noted; one had N0 and three had N+ lymph node invasion, and 4/7 had distant metastasis. CT scanning was available, and the mean size was 62.7 ± 35.9 mm in the pandemic period, while it was 70.2 ± 32 mm during the pre-pandemic period (*p* = 0.6078). Endoscopy was performed in 5 of 17 small bowel malignant tumors during both periods, and abdominal ultrasound was conducted in 3 of 17 cases in both periods. The limited role of both investigations was explained by the fact that most tumors were large and mostly diagnosed by CT scan.

In colorectal cancers, a slightly but statistically significant difference regarding age was noted (67.9 years during the pandemic period versus 66.3 years during the pre-pandemic period, *p* = 0.0141); the gender was similar. Almost all tumors were carcinomas (adenocarcinoma being noted in 93.3–96.4%), with similar grading. Slightly more stage II cancers were also recorded (25.9 versus 22.5%, *p* = 0.0451), although no differences between T1–4, N, and M staging were noted. Contrary to other studies, a slightly lower percentage of vascular invasion was noted during the pandemic (16.2 versus 25.5%, *p* = 0.0034). The location was also similar, but fewer Borrmann I and more Borrmann III tumors were noted during the pandemic. A slightly higher proportion of tumors was observed at CT scan during the pandemic than in the similar pre-pandemic period (83.4 versus 72.7, *p* = 0.0011). The complication rate was higher during the pandemic, but the difference was mostly noted for stenosing tumors at colonoscopy, while occlusive, bleeding, and perforated tumors were similar. Surgical rates were similar, but more polypectomies for T1 tumors were noted. Perioperative mortality was higher during the pandemic (9.3 versus 5.3%, *p* = 0.0189), and emergency surgery rates and the percentage of invaded margins were similar (Table 3).

### 3.2. Overall Survival in Gastric, and Colorectal Tumors

**Figure 2:**
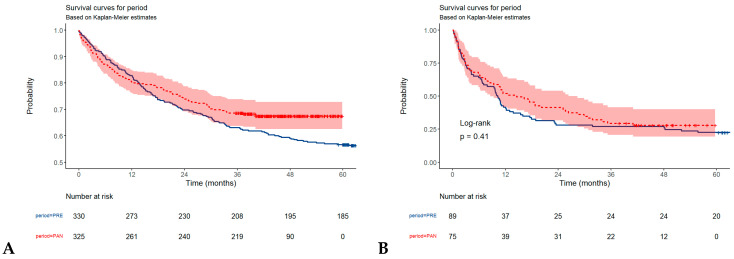
Overall survival in patients with colorectal cancer (**A**), and gastric cancer (**B**).

We analyzed the overall survival in patients with gastric and colorectal carcinomas during the pre-pandemic and pandemic periods, respectively. Between January 2018 and December 2021, 1058 patients were diagnosed with CRC and 419 were diagnosed with gastric cancer. Among CRC patients, 258 were excluded for the lack of data (no death date, T stage, N stage) and 145 patients were excluded as they were dead perioperatively, leaving 655 patients for the analysis. Among gastric cancer patients, 164 patients were included in this analysis after excluding 255 patients for the lack of data. The OS was surprisingly higher during the pandemic, with an HR of 0.78 (95%CI 0.60–1.00, *p* = 0.051) in CRC cancer patients and 0.86 (95%CI 0.60–1.23, *p* = 0.412) in the case of gastric cancer patients (Figure 2A,B). This suggests that the risk of death during the pandemic period was 22% lower compared to the pre-pandemic period for CRC patients, but the result is not statistically significant. The same result, but without statistical significance, was obtained for gastric patients: the risk of death during the pandemic period was 14% lower compared to the pre-pandemic period.

Median OS during the pandemic (excluding perioperative mortality) was not available (the survival curve does not drop below ½ during the observation period) for colorectal cancer and was 14.6 (95%CI, 8.5–28.4) months for gastric cancer. Median OS during the pre-pandemic period (excluding perioperative mortality) was undefined (the survival curve does not drop below ½ during the observation period) for colorectal cancer and was 10.2 (95%CI, 7–13) months for gastric cancer.

**Figure 3:**
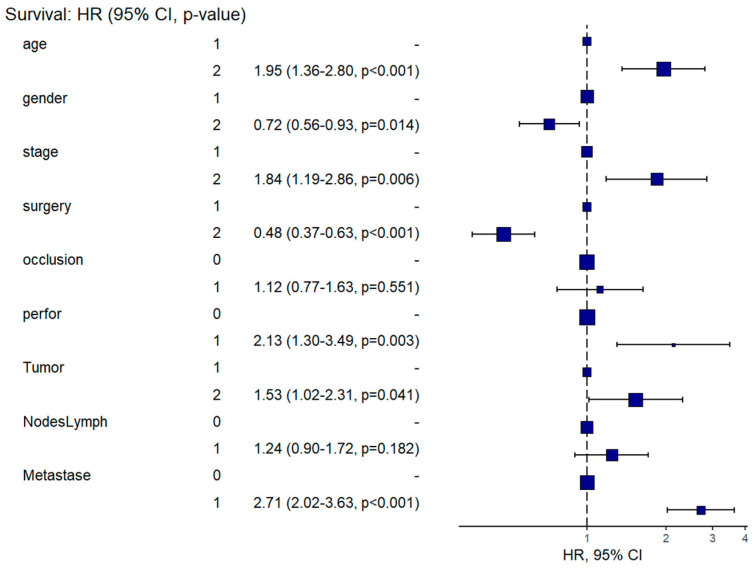
Hazard regression plot with adjusted analysis for overall survival in colorectal cancer. CI, confidence interval; HR, hazard ratio.

The overall survival of CRC patients was influenced by age over 60 years, advanced stage (III + IV), radical surgery, tumor perforation, deep tumor invasion (T3 + T4), the presence of positive lymph nodes (N+), and the presence of metastases (M1) (Table 4, Figure 3). Patients aged 60 or older have almost twice the risk of mortality compared to those under 60, which remains significant even after adjusting for other factors in the multivariate analysis (HR = 1.95, 95%CI: 1.36–2.80, *p* < 0.001), indicating that age is an independent risk factor for worse OS. Although the univariate analysis did not show a statistically significant difference, the multivariate analysis revealed that the female gender was associated with a 28% lower risk of mortality compared to males (HR = 0.72, 95%CI: 0.56–0.93, *p* = 0.014), possibly because of other confounding factors.

Higher TNM stages (III + IV) were strongly associated with worse OS in both univariate and multivariate analyses, but the hazard ratio was attenuated in the multivariate model, suggesting that part of the effect is mediated by other factors, such as tumor characteristics or treatment approaches. Advanced tumor depth (T3 + T4) was associated with worse OS. Although the effect size decreases in the multivariate model (HR = 1.53, 95%CI: 1.02–2.31, *p*-value = 0.041), it remains statistically significant, indicating that tumor invasion is an independent risk factor. While lymph node involvement was significantly associated with worse OS in univariate analysis (HR = 2.23, 95%CI: 1.73–2.88, *p*-value < 0.001), this association loses significance in the multivariate model (*p* > 0.05). This suggests that the effect of lymph node status may be mediated by other factors, such as metastases or TNM stage. The presence of metastases was the strongest predictor of poor OS (HR = 4.34, 95%CI: 3.36–5.61, *p* < 0.001), with patients having more than twice the risk of mortality even after adjustment (HR = 2.71, 95%CI: 2.02–3.63, *p* < 0.001). The attenuation of the hazard ratio in the multivariate model suggests that some of this effect is influenced by other covariates.

Radical surgery was associated with significantly better OS, even after adjusting for other variables (*p* < 0.001). The adjusted HR indicates a 52% reduction in mortality risk for patients undergoing radical surgery. The presence of occlusion does not appear to have a significant impact on OS in either univariate or multivariate analyses (*p* > 0.05), while perforation significantly increases the risk of mortality, and this association is even stronger in the multivariate model (HR = 2.13, 95%CI: 1.30–3.49, *p* = 0.003).

## 4. Discussion

Admission rates were significantly decreased for esophageal, gastric, and colorectal cancers, with a more severe decline for esophageal and eso-cardial tumors (30.3%) and gastric tumors (27.6%), whereas for colorectal cancers, the decline was less severe (17.3%). The decline may be explained by a combination of factors, such as pandemic-related restrictions (lockdown), the fear effect (patients were reluctant to come to the hospitals), and restrictions regarding endoscopies (especially for upper digestive endoscopy, an aerosol-induced procedure). The decline was more pronounced in studies focused on the first months of the pandemic, with a reduction of 45.5% for CRC and 46.5% for gastric cancer during January–May 2020 in Philadelphia [58], and a decline of newly diagnosed cancers by 44.2%, with 43.5% fewer new colorectal carcinomas from March to June 2020 in another US-based study [15]. Studies focused on a larger segment of pandemic time have noted a smaller but significant reduction in newly diagnosed cancers, with 26.87 and 13.8% declines for gastric carcinoma, 13.47 and 4.3% for CRC, and 3.6% for esophageal carcinoma in two Japan studies covering 2020 as compared to the pre-pandemic period [7,13]. Similar data were obtained in two national, US-based studies that compared 2020 with the 2018–2019 period: all newly diagnosed cancers decreased by 12.8% as compared to 2018 and by 14.7% as compared to 2019 (the decrease was 15.4 and 16.6% for gastric carcinoma, 14 and 14.9% for CRC, and 9.4 and 13.1% for esophageal carcinoma) [18]. Also, there was a decrease between 13 and 23% for all newly diagnosed cancers, associated with a reduction of colonoscopies by 45% and of CT scans by 10% [59]. A rebound was recorded after June 2020, but without attaining previous levels [59]. Another study from Quebec, Canada, found fewer diagnosed colorectal (by 20.5%), gastric (by 22.6%), and esophageal carcinomas (by 16.6%) with a similar staging [60]. In a multicenter study performed in five European countries, in 4.5%, a treatment delay was noted, although no more than 3 months [8]. The reduction in newly diagnosed cancers was heterogeneous, as differences were noted between cancer locations [7,13,14,18,26,35,60,61,62] and in older patients [59].

In our study, no staging differences in esophageal, eso-cardial, and small bowel cancers were recorded. During the pandemic, more stage III tumors in gastric cancers (40.3 versus 31.2%) and slightly more stage II colorectal cancers (25.9 versus 22.5%) were noted, although the differences between T1–4, N1–3, and M stages were not statistically significant. The literature data reported conflicting results, with most studies assessing colorectal carcinomas. For esophageal carcinoma, no stage differences were noted in one study [13], while another two studies found more advanced disease during the pandemic [7,18], with an OR for stage IV versus stages I–III of 1.076 (1.014–1.142) [18]. Two CT scan-based studies in Italy and Japan have shown more advanced stage for all cancers [17,63], with an OR of 1.56 for more advanced disease, 1.84 for lymph node positivity (N+), and 2.09 for metastatic disease [17], as well as also more stage II esophageal cancers [63]. For gastric carcinomas, two studies from Japan assessing the period between January and December 2020 have shown 35.5% and 15.3% fewer stage I gastric carcinomas, while stage IV had no statistically significant difference [7,13], and another study has shown 20.1% fewer gastric carcinomas, but with similar stages [64]. The difference in stage I gastric cancers in Japan may be related to the decreased screening procedures. In an Italian study of gastric carcinoma at nine centers between January 2019 and November 2020, the cTNM staging was similar [33]. In a USA-based study, the odds ratio for stage IV was 1.129 for gastric carcinoma [18].

For CRC, a study in the USA on four cancer sites has shown that, from March to June 2020, 13% more cases of stage IV CRC were noted, and unadjusted OR for stage IV was 1.27 (however, adjusted OR was not statistically significant) [15]. In a national, USA-based study of the National Database of Cancer (70% of all cancers), a global reduction of newly diagnosed cancers was noted in the first pandemic year for CRC (15.6%); the decrease was more significant for early stages than for advanced stages (19.3 and 12.3%, respectively) [61], while another national, US-based study found that in 2020 more stage IV CRCs were diagnosed (12.9 versus 4.5% in 2015–2019), with the same finding for stage IV rectal carcinoma (2.2 versus 0.8%) [28]. The odds ratio for stage IV in the USA was 1.053 [18]. Two small studies from the USA and one from Brazil also found similar TNM staging [31,37,62]. In a 2020–2019 study in Sao Paolo, Brazil, screening procedures for colorectal cancer were reduced by 45%, diagnosis was reduced by 35%, and surgery for tumors was reduced by 15% as compared to 2018–2019, but no differences were noted regarding the presence of metastases (M1), lymph nodes (N+) or early (I–II) versus advanced stage (III–IV) [10]. In Japan, there was a decrease in stages 0 (by 32.89%), 1 (by 34.04%), and II (by 35.22%), while stage III increased by 68.42% and stage IV was similar [13], while three other studies showed a decrease in early (0/I) stages of CRC (24.2 versus 26.9%) [22], with a 7.2% increase in stage IV in 2020 [30] and a small but statistically significant difference for more advanced versus early cases diagnosed in CRC (34.4 versus 65.6, respectively) [7]. A rebound was noted in 2021 for newly diagnosed and early-stage cancers [30]. In a Morocco study including all digestive cancers (except for esophagus) operated on in 2020 and 2021 as compared to the pre-pandemic year, more cancers were diagnosed in stages III–IV, but with similar complications and surgical procedures [14]. In a 15-month pandemic period study in Turkey, T3, T4, and N+ tumors were similar [19]. In an Italian study in eight centers comparing 2020 to the pre-pandemic period, there were 63% stage I–III colorectal tumors during the pandemic and 78% during the pre-pandemic period, with more stage IV cases during the pandemic (37 versus 22%) [21]. The stage was similar in a Spanish study [27] and in two studies from the Netherlands [20,34]. In the UK, two studies showed more T4 [25,36] and M1 tumors [36] but similar N+ stages [25,36]. In Serbia, a study of surgical management in CRC during the pandemic has shown a higher percentage of T4b and IIC tumors (12.2 versus 3.3%, and 10.2 versus 1.3%, respectively) [23]. In Israel, a study comparing 2020 and 2019 admissions for CRC has shown only small and not statistically significant stage differences [29]. In Korea, a study regarding the 2020–2021 period has found, surprisingly, a reduction of stage III+IV CRC during the pandemic (45.3 versus 51.3%), possibly related to the absence of both lockdown and hospital restrictions during 2020–2021 [32].

The absolute number of surgical procedures was reduced during the pandemic as a result of various restrictions and patients’ fear of hospitals and admissions [9,10,22,56]. The resection rate was decreased during the pandemic in an Italian study of gastric carcinoma patients admitted at nine centers between January 2019 and November 2020 (64.2% versus 88.7%), with more cases being converted from laparoscopic to open surgery (23.7 versus 8%) [33] and an increased percentage of patients presenting with ileus. Also, stoma procedures were recorded in two studies from the Netherlands in the second trimester of 2020 [20,34]. However, a London-based study comparing CRC patients admitted in 2020–2021 compared to 2019 found no statistically significant differences regarding the number of colectomies and pathological aspects [35].

In our study, for gastric cancers, the rate of complications, emergency surgery rates, total and emergency surgeries, perioperative mortality, and the percentage of invaded margins were similar. For CRC, surgical rates were the same, but more polypectomies for T1 tumors were noted; perioperative mortality was almost two-fold higher during the pandemic, while emergency surgery rates and the percentage of invaded margins were similar. More stenosing tumors were noted during the pandemic, but the rates of occlusions and perforations were similar. Emergency presentations and surgery, especially in CRC, were found more frequently during the pandemic in several studies [19,25,27,47,65], but not in others [66]. In a 15-month pandemic period study in Turkey, obstructive CRC was found in 69.2% as compared to 47.2% in the similar pre-pandemic period [19]. In a Spanish study of patients admitted between September 2020 and March 2021 compared to the September 2019–January 2020 period, more cases of complicated colorectal cancers were recorded (14.6 versus 10.4%), although staging was similar [27]. Two studies from the UK have shown an increased emergency presentation for CRC (36 versus 28.6%, and 21 versus 16%, respectively), with more frequent emergency surgery [25,36].

A lower percentage of colorectal tumors with lymphatic invasion was recorded in our study during the pandemic. In a Brazilian study, more aggressive tumors (with higher vascular, lymphatic, and perineural invasion) were found during the pandemic period [31]; similar data regarding more cancers with lympho-vascular and perineural invasion during the pandemic were obtained in a Romanian study [48].

The potential delay in cancer diagnosis and surgery may decrease overall survival [65]. In our study, the overall survival of gastric and CRC (but without statistical significance) patients was surprisingly higher during the pandemic, although a clear explanation has not emerged. The OR for survival was 1.24 for gastric cancer patients and 1.36 for colorectal cancer patients, although the statistical significance was only obtained for colorectal cancer. We have no explanation for the higher survival during the pandemic period, but we expected a lower survival for the cancer patients diagnosed during the pandemic as a result of delayed diagnosis, possible more advanced staging, less access to oncological therapy and surgery, and the risk of COVID-19 infection for cancer patients with altered immune status. The data regarding the survival of patients with solid tumors during the pandemic were scarce, with some studies pointing to a decreased survival [67,68,69,70] while others showed no significant difference [71,72].

In Romania, four studies have evaluated the surgical management of digestive cancers during the pandemic; two included CRC [46,48], one included only colon cancer [47], and one included rectal carcinoma [16]. A more advanced stage was noted in the pandemic period in all studies, with stage IV rectal carcinoma in 25.4% of cases in 2022 as compared to 12.5% in 2020 and 9.1% in 2019 [16]; metastatic disease in 26.8% versus 12.5% [48]; more emergency admissions; and more colostomies during the pandemic period [48]. Emergency admissions were higher in one study [48], but a decrease in the second pandemic year was also recorded [16]. Perioperative mortality was similar in both periods [16,46,47,48]. In contrast, we found a higher perioperative mortality for CRC, but not higher rates of metastatic disease.

The reduction of admissions for esophageal and gastrointestinal cancers in our study can be explained mainly by the fear effect generated by the pandemic, which limited the diagnosis of cancers in symptomatic patients; the screening programs for gut tumors in Romania were underdeveloped in both the pre-pandemic and pandemic periods. Small differences found regarding more stage III gastric tumors, more stage II colorectal tumors, and a higher proportion of stenosing esophageal and colorectal tumors could suggest that significantly more newly diagnosed (and potentially more advanced) gut tumors would appear after 2022, thus impacting the oncological and surgical care of cancer patients. The impact of the pandemic on perioperative mortality was significant only for colorectal cancers, with more than double mortality during the pandemic period; the explanations may be related to the more locally advanced cancers or healthcare changes during the pandemic (from the effect of protective gear to the accuracy of surgical procedures, as well as postoperative care difficulties).

Future health crises related to pandemics need to be taken into consideration. We believe that our research has shown how important access to care can be in such situations, and we need to address key aspects to ensure the quality of oncological care. Effective and streamlined screening programs can eliminate inequalities and ensure access to early intervention for many at-risk populations [73,74,75,76,77]. To attain this goal, close collaboration with non-governmental organizations, as well as state actors and local authorities, should be maintained; financial, logistical and emotional support can thus be provided for broader cancer populations [73]. All medical actors should provide public education and clear communication to cancer patients. Cancer patients will then be properly informed regarding virtual consultations, adjustment to treatment protocols, as well as general safety regulations while accessing healthcare [73,74,75,76,77]. Hospital structures need to be flexible and resilient to pandemics, increasing the capacity of cancer treatment centers and stockpiling critical medications [75]. Designating medical facilities for specific needs should ensure that no oncology service is disrupted, by applying risk-based triage for cancer treatments, flexible scheduling and treatment planning, as well as effective protective measures for both patients and medical personnel [74].

The main limitations of this study are related to its single-center nature, the sample size (especially for esophageal and eso-cardial cancers), the statistical power, and the presence of unmeasured confounders (e.g., oncological treatment, the time interval between symptoms onset and presentation and between first presentation and definitive diagnosis, the changes in healthcare access, and prioritization of patients), which might have influenced the result. Future studies with larger sample sizes might help clarify whether the observed trend of better survival for patients diagnosed during the pandemic reflects a true protective effect of OS or was induced by a delayed presentation during the COVID-19 pandemic, which may be reflected by a potentially higher number of newly diagnosed and advanced cases of gut tumors after 2022.

## 5. Conclusions

In our study, no staging differences in esophageal, eso-cardial, and small bowel cancers were recorded, but more stage III tumors in gastric cancers and slightly more stage II colorectal cancers were noted. Surgery was similar, but perioperative mortality was almost two-fold higher for CRC during the pandemic, and more stenosing tumors were noted with the same rate of occlusions and perforations. A lower percentage of colorectal tumors with lymphatic invasion was recorded in our study during the pandemic. The overall survival of CRC and gastric cancer patients was surprisingly higher during the pandemic, but without statistical significance compared to the pre-pandemic period; a clear explanation has not emerged. Future studies including patients admitted after 2022 can better assess the impact of the pandemic on gut cancer admissions and treatment, as well as the influence on overall survival.

## Figures and Tables

**Table 1:** Esophageal and eso-cardial tumor characteristics.

	PANDEMIC −46 pts-	PRE-PANDEMIC −66 pts-	*p*-Value
-Age (mean ± stdev, Min–Max)	61.8 ± 7.8 (35–77)	61.8 ± 11.1 (32–86)	0.9970
-Gender Male/Female (%Male)	35/11 (76.1)	60/6 (90.9)	0.0374
-pathological type			
Carcinoma			
-adenocarcinoma	7 (20.6)	11 (20.7)	0.9851
-squamous	27 (79.4)	42 (79.3)	
-carcinoma NOS	12	13	
GIST	1	2	
Sarcoma	1	0	
-grading (carcinoma)			
G1	0 (0)	5 (12.2)	0.1551
G2	8 (32)	12 (29.3)	0.9144
G3	15 (60)	23 (56.1)	0.8055
G4	2 (8)	1 (2.4)	0.3824
-staging (carcinoma)			
I	0 (0)	2 (3.6)	0.4114
II	4 (10)	10 (18.2)	0.3152
III	18 (45)	21 (38.2)	0.4249
IV	18 (45)	22 (40)	0.5291
NA	6	11	0.5999
-T stage (carcinoma)			
T1	0 (0)	2 (4.8)	0.4114
T2	2 (5.6)	4 (9.5)	0.6934
T3	17 (47.2)	12 (57.1)	0.0281
T4	17 (47.2)	24 (28.6)	0.9489
Tx	10	24	0.1008
-N stage (carcinoma)			
N0	13 (35.1)	17 (34)	0.9123
N+	24 (64.9)	33 (66)	
Nx	9	16	0.5593
-M stage (carcinoma)			
M0	24 (60)	33 (60)	1.0000
M1	16 (40)	22 (40)	
Mx	6	11	0.5999
-Location			
Superior	17 (42.5)	20 (32.8)	0.4620
Middle	10 (25)	22 (36.1)	0.1842
Inferior	5 (12.5)	8 (13.1)	0.8389
Eso-cardial	8 (20)	11 (18)	0.9199
Unknown	6	5	0.3441
-Borrmann type			
I	10 (35.7)	12 (26.1)	0.6415
II	13 (46.4)	31 (67.4)	0.0481
III	0 (0)	1 (2.2)	0.6552
IV	5 (17.9)	2 (4.3)	0.1135
Unknown	20	20	0.1540
-CT scan No (%)	36 (76.6)	42 (60)	0.1008
Visible tumor	33 (91.7)	32 (76.2)	0.0792
-Surgery			
Radical	2 (4.3)	5 (7.6)	0.4928
Palliative gastrostomy	18 (39.1)	12 (18.2)	0.0156
-Esophageal stent	4 (8.7)	3 (4.5)	0.3960
-Complications			
Stenosis	35 (76.1)	37 (56.1)	0.0317
Bleeding	3 (6.5)	4 (6.1)	0.9210
Perforation	1 (2.2)	0 (0)	0.3687

NOS = not otherwise specified, GIST = gastrointestinal stromal tumors.

**Table 2:** Gastric tumor characteristics.

	PANDEMIC −176 pts-	PRE-PANDEMIC −243 pts-	*p*-Value
-Age (mean ± stdev, Min–Max)	67.2 ± 12.4 (26–88)	67.2 ± 11.7 (35–92)	0.9709
-Gender Male/Female (%Male)	107/69 (60.8)	153/90 (63)	0.6518
-pathological type			
Carcinoma No (%)			
-adenocarcinoma	97 (91.5)	126 (88.1)	0.7891
-adenosquamous	1	1	
-poorly cohesive	25	31	
-mixed	14	15	
-carcinoma NOS	24	41	
Lymphoma	5	10	
Neuroendocrine	1	3	
GIST	7	16	
Sarcoma	2	0	
-grading (carcinoma)			
G1	10 (10.5)	1 (0.8)	0.0233
G2	34 (35.8)	48 (40)	0.0411
G3	34 (35.8)	28 (23.3)	0.3538
G4	2 (2.1)	5 (4.2)	0.2640
-staging (carcinoma)			
I	7 (4.4)	7 (4.1)	0.8982
II	3 (1.9)	9 (5.3)	0.1190
III	64 (40.3)	53 (31.2)	0.0300
IV	85 (53.5)	101 (59.4)	0.3365
Unknown	8	18	0.0901
-T stage (carcinoma)			
T1	3 (2.34)	0 (0)	0.2721
T2	10 (7.81)	16 (10.8)	0.1381
T3	63 (49.22)	57 (38.5)	0.5746
T4	56 (43.75)	47 (31.8)	0.5994
Tx	44	78	0.0032
-N stage (carcinoma)			
N0	41 (30.1)	33 (26.6)	0.3053
N1	33 (24.3)	32 (25.8)	0.7743
N2	28 (20.6)	28 (22.6)	0.6964
N3	34 (25)	31 (25)	1.0000
N+	95 (69.9)	91 (73.4)	0.5284
Nx	40	74	
-M stage (carcinoma)			
M0	76 (48.4)	76 (37.6)	0.3166
M1	81 (51.6)	101 (50)	
Mx	12	17	0.5718
-Lymphatic/vascular/perineural ((carcinoma)			
L1	20 (36.4)	21 (48.8)	0.2155
V1	21 (38.2)	29 (63)	0.0138
PN1	33 (60)	27 (56.3)	0.7003
-Location			
Antro-pyloric	54 (34.4)	48 (25.0)	0.3623
Corporeal	71 (45.2)	74 (38.5)	0.9555
Cardial	40 (25.5)	51 (26.6)	0.2506
Stump	7 (4.5)	5 (2.6)	0.5193
-Borrmann type			
I	19 (16.8)	16 (10.7)	0.2726
II	58 (51.3)	74 (49.3)	0.6388
III	30 (26.5)	34 (22.7)	0.8180
IV	18 (15.9)	25 (16.7)	0.5901
-CT scan	110 (62.5)	123 (50.6)	0.0160
Visible tumor	76 (69.1)	70 (56.9)	0.0559
-Complications			
Bleeding	37 (23)	46 (21.50)	0.5959
Stenosis	32 (19.9)	33 (15.42)	0.2005
Perforation	2 (1.2)	4 (1.87)	0.6665
-Surgery (%)	96 (54.6)	122 (50.2)	0.3803
Radical	68 (38.6)	85 (35)	0.4430
Palliative gastrectomy	2 (1.1)	4 (1.6)	0.6665
Complications	20 (11.4)	19 (7.8)	0.2202
Laparotomy	6 (3.4)	14 (5.8)	0.2703
-Perioperative mortality (%)	12/96 (12.5)	13/130 (10.7)	0.5358
-Invaded margins	14/60 (23.3)	26/77 (33.8)	0.1847
-Emergency surgery	31/96 (32.3)	32/122 (26.2)	0.3276

NOS = not otherwise specified, GIST = Gastrointestinal stromal tumors, L1 = lymphatic invasion present, V1 = vascular invasion present, PN1 = perineural invasion present.

**Table 3:** Colorectal tumor characteristics.

	PANDEMIC −479 pts-	PRE-PANDEMIC −579 pts-	*p*-Value
-Age (mean ± stdev, Min–Max)	67.9 ± 10.4 (29–91)	66.3 ± 10.6 (23–88)	0.0141
-Gender M/F (%M)	286/193 (59.7)	349/230 (60.3)	0.8509
-pathological type			
Carcinoma No (%)	476 (99.4)	575 (99.3)	0.8175
-adenocarcinoma	447 (93.3)	558 (96.4)	
-adenosquamous	3 (0.6)	1 (0.2)	
-mucinous	0 (0)	2 (0.4)	
-mixed	21 (4.4)	77 (13.3)	
-carcinoma NOS	29 (6)	18 (3.1)	
Lymphoma	0 (0)	0 (0)	
Neuroendocrine	1 (0.2)	3 (0.5)	
Sarcoma	0 (0)	1 (0.2)	
GIST	2 (0.4)	0 (0)	
-grading (carcinoma)			
G1	51 (13.8)	45 (10.0)	0.0904
G2	240 (65.0)	298 (65.9)	0.7495
G3	70 (19.0)	99 (21.9)	0.2927
G4	7 (1.9)	10 (2.2)	0.7483
-staging (carcinoma)			
0	4 (0.9)	1 (0.2)	0.1615
I	55 (12.1)	64 (11.8)	0.8937
II	118 (25.9)	122 (22.5)	0.0451
III	155 (34.0)	193 (35.5)	0.6081
IV	124 (27.2)	163 (30)	0.3257
Unknown	23 (4.8)	36 (6.2)	0.3190
-T stage (carcinoma)			
Tis	1 (0.2)	0 (0)	0.4679
T0	3 (0.7)	1 (0.2)	0.3039
T1	18 (4)	15 (3.1)	0.4350
T2	71 (15.9)	84 (17.2)	0.5755
T3	232 (51.9)	253 (51.8)	0.9880
T4	122 (27.3)	134 (27.5)	0.9393
Tx	32 (6.7)	92 (15.9)	<0.0001
-N stage (carcinoma)			
N0	211 (50.2)	231 (48)	0.5270
N1	138 (32.9)	162 (33.7)	0.7768
N2	70 (16.7)	86 (17.9)	0.6212
N3	1 (0.2)	1 (0.2)	0.9247
N+	209 (48.8)	249 (49.5)	0.5270
Nx	59 (12.3)	99 (17.1)	0.0305
-M stage (carcinoma)			
M0	338 (73.5)	376 (69)	0.1291
M1	122 (26.5)	168 (30.8)	
Mx	19 (4.1)	35 (6.4)	0.4990
-Lymphatic/vascular/perineural ((carcinoma)			
L1	25 (7.9)	17 (4.8)	0.0931
V1	51 (16.2)	91 (25.5)	0.0034
PN1	76 (24.1)	83 (23.2)	0.7894
-Location			
Rectum	176(37.1)	222 (38.6)	0.6235
Sigmoidum	135 (28.5)	158 (27.5)	0.7187
Descending	49 (10.3)	40 (7)	0.0519
Transverse	23 (4.9)	40 (7)	0.1555
Ascending	61 (12.9)	75 (13)	0.9334
Caecum	30 (6.3)	40 (7)	0.6854
Not Defined	5	4	0.5366
-Borrmann type			
I	104 (28.7)	85 (44.3)	0.0003
II	196 (54.1)	94 (49)	0.2452
III	36 (9.9)	3 (1.6)	0.0014
IV	26 (7.2)	10 (5.2)	0.3717
Not Defined	117	387	<0.0001
-CT scan	236 (49.3)	268 (46.3)	0.3337
Visible tumor	196 (83.4)	189 (72.7)	0.0011
-Complications No (%)	249 (52.0)	248 (42.8)	0.0030
Bleeding	75 (15.7)	104 (18)	0.3200
Stenosis	96 (20)	37 (6.4)	<0.0001
Occlusion	58 (12.1)	70 (12.1)	0.9926
Perforation	18 (3.8)	23 (4)	0.8572
Fistula	1 (0.2)	9 (1.6)	0.5556
Abscess	0 (0)	4 (0.7)	0.1769
-Surgery/endoscopic resection			
Polypectomy	9 (1.9)	1 (0.2)	0.0173
Radical	312 (65.1)	378 (65.2)	0.3555
Palliative	51 (10.6)	50 (8.6)	0.1397
Laparotomy	0 (0)	1 (0.2)	0.6382
Biopsy	2 (0.4)	3 (0.5)	0.9344
No	105 (21.9)	146 (25.2)	0.2101
-Perioperative mortality	34/365 (9.3)	23/432 (5.3)	0.0189
-Invaded margins	13/299 (4.4)	13/353 (3.7)	0.6657
-Emergency surgery	128/363 (35.3)	134/428 (31.3)	0.2393

NOS = not otherwise specified, GIST = Gastrointestinal stromal tumors, L1 = lymphatic invasion present, V1 = vascular invasion present, PN1 = perineural invasion present.

**Table 4:** Correlation between overall survival and clinical variables in univariate and multivariate analysis for patients with CRC (N = 655 patients).

Factor	Univariate Analysis		Multivariate Analysis	
	Hazard Ratio (95%CI)	*p*-Value	Hazard Ratio (95%CI)	*p*-Value
Age (years) ≥ 60 vs. <60	1.99 (1.39–2.84)	<0.001	1.95 (1.36–2.80)	<0.001
Gender, Female vs. Male	0.85 (0.65–1.09)	0.201	0.72 (0.56–0.93)	0.014
TNM stage, III + IV vs. I + II	3.80 (2.81–5.13)	<0.001	1.84 (1.19–2.86)	0.006
Surgery, radical vs. palliative	0.43 (0.33–0.56)	<0.001	0.48 (0.37–0.63)	<0.001
Occlusion, yes vs. no	1.07 (0.74–1.55)	0.711	1.12 (0.77–1.63)	0.551
Perforation, yes vs. no	1.88 (1.17–3.04)	0.01	2.13 (1.30–3.49)	0.003
Tumor, T3 + T4 vs. T1 + T2	2.42 (1.67–3.50)	<0.001	1.53 (1.02–2.31)	0.041
Lymph Nodes, N+ vs. N0	2.23 (1.73–2.88)	<0.001	1.24 (0.9–1.72)	0.182
Metastases, M1 vs. M0	4.34 (3.36–5.61)	<0.001	2.71 (2.02–3.63)	<0.001

## Data Availability

The data presented in this study are available on request from the corresponding author.

## Supplementary Materials

The following supporting information can be downloaded at: https://www.mdpi.com/article/10.3390/healthcare13070805/s1, Table S1. Definition of category T, N, and M for esophageal carcinoma [52]. Table S2. Clinical (cTNM) staging for esophageal carcinoma [52]. Table S3. Definition of category T, N, and M for gastric carcinoma [53,54]. Table S4. Clinical (cTNM) and pathological (pTNM) staging for gastric carcinoma [53,54]. Table S5. Definition of T, N, and M categories for colorectal carcinoma [55]. Table S6 Clinical and pathological staging for colorectal carcinoma [55].
