# Supplementary material for: Impact of the COVID-19 Pandemic on Gut Cancer Admissions and Management: A Comparative Study of Two Pandemic Years to a Similar Pre-Pandemic Period
Source: Healthcare (Basel). 2025 Apr 3;13(7):805. doi: 10.3390/healthcare13070805 (PMC11988892; doi:10.3390/healthcare13070805)
Supplement: Supplementary file 1 [file healthcare-13-00805-s001.zip › healthcare-3457100-supplementary.pdf]

Supplementary Table S1 Definition of category T, N, and M for esophageal carcinoma [52]

T category	
Tis	High-grade dysplasia, defined as malignant cells confined by the basement membrane
T1	Tumor invades the lamina propria, muscularis mucosae, or submucosa
T1a*	Tumor invades the lamina propria or muscularis mucosae
T1b*	Tumor invades the submucosa
T2	Tumor invades the muscularis propria
T3	Tumor invades adventitia
T4	Tumor invades adjacent structures.
T4a*	Tumor invades the pleura, pericardium, azygos vein, diaphragm, or peritoneum.
T4b*	Tumor invades other adjacent structures, such as the aorta, vertebral body, or trachea.
N category	
N0	No regional lymph node metastasis
N1	Metastasis in 1–2 regional lymph nodes
N2	Metastasis in 3–6 regional lymph nodes
N3	Metastasis in 7 or more regional lymph nodes
M category	
M0	No distant metastasis
M1	Distant metastasis

Supplementary Table S2 Clinical (cTNM) staging for esophageal carcinoma [52]

Stage	Squamous cell carcinoma	*Stage*	Adenocarcinoma
0	Tis N0 M0	0	Tis N0 M0
I	T1 N0–1 M0	I	T1 N0 M0
II	T2 N0–1 M0	IIA	T1 N1 M0
	T3 N0 M0	IIB	T2 N0 M0
III	T3 N1 M0	III	T2 N1 M0
	T1–3 N2 M0		T3–4a N0–1 M0
IVA	T4 N0–2 M0	IVA	T1–4a N2 M0
	T1–4 N3 M0		T4b N0–2 M0
			T1–4 N3 M0
IVB	T1–4 N0–3 M1	IVB	T1–4 N0–3 M1

Supplementary Table S3 Definition of category T, N, and M for gastric carcinoma [53,54]

T category	
Tis	High-grade dysplasia, defined as malignant cells confined by the basement membrane
T1	Tumor invades the lamina propria, muscularis mucosae, or submucosa
T1a	Tumor invades the lamina propria or muscularis mucosae
T1b	Tumor invades the submucosa
T2	Tumor invades the muscularis propria
T3	Tumor invades adventitia
T4	Tumor invades serosa/adjacent structures.
T4a	Tumor invades the serosa (visceral peritoneum)
T4b	Tumor invades adjacent structures (spleen, transverse colon, liver, diaphragm, pancreas, abdominal wall, adrenal gland, kidney, small intestine and retroperitoneum)
N category	
N0	No regional lymph node metastasis
N1	Metastasis in 1–2 regional lymph nodes
N2	Metastasis in 3–6 regional lymph nodes
N3	Metastasis in 7 or more regional lymph nodes
-N3a	Metastasis in 7–14 regional lymph nodes

 https://doi.org/10.3390/healthcare13070805

-N3b	Metastasis in 15 or more regional lymph nodes
M category	
M0	No distant metastasis
M1	Distant metastasis

Supplementary Table S4 Clinical (cTNM) and pathological (pTNM) staging for gastric carcinoma [53,54]

cTNM	Clinical staging	*pTNM*	Pathological staging
0	Tis N0 M0	0	Tis N0 M0
I	T1 N0 M0	IA	T1 N0 M0
	T2 N0 M0	IB	T2 N0 M0
			T1 N1 M0
IIA	T1-2 N0 M0	IIA	T3 N0 M0
			T2 N1 M0
			T1 N2 M0
IIB	T3-4a N0 M0	IIB	T4a N0 M0
			T3 N1 M0
			T2 N2 M0
			T1 N3 M0
III	T3-4a N+ M0	IIIA	T4b N0 M0
			T4a N1-2 M0
			T3 N2 M0
			T2 N3a M0
		IIIB	T4b N1 M0
			T4a N2 M0
			T3 N3a M0
			T1 N1b M0
IVA	T4b Nx M0	IV	Tx Nx M1
IVB	Tx Nx M1		

Supplementary Table S5 Definition of T, N, and M categories for colorectal carcinoma [55]

T category	
Tis	Carcinoma *in situ*, limited to intraepithelial or invasive lamina propria
T1	Tumor invades the submucosa
T2	Tumor invades the muscularis propria
T3	Tumor penetrating the muscularis propria and arriving at colorectal fat tissue
T4	Tumor directly invades other organs or structures.
T4a	Tumor penetrating visceral peritoneum
T4b	Tumor directly invading or adhering to other organs or structures.
N category	
N0	No regional lymph node metastasis
N1	Metastasis in 1–2 regional lymph nodes
-N1a:	1 lymph node metastases
-N1b	2-3 lymph nodes metastases
-N1c	Although there was no regional lymph node metastasis, TDs were submucosal, mesangial, or peritoneum-covered para-colorectal tissue.
N2	Metastasis in 4or more regional lymph nodes
-N2a	Metastasis in 4–6 regional lymph nodes
-N2b	Metastasis in 7 or more regional lymph nodes
M category	
M0	No distant metastasis
M1	Distant metastasis or distant lymph nodes
-M1a	Metastasis is limited to one organ or site (*e.g.*, liver, lung, ovary, and extra-regional lymph node metastases)

-M1b	More than one organ or site<br>Peritoneal metastases with or without metastasis of other organs1

Supplementary Table S6 Clinical and pathological staging for colorectal carcinoma [55]

cTNM/pTNM	Pathological staging
0	Tis N0 M0
I	T1 N0 M0
	T2 N0 M0
IIA	T3 N0 M0
IIB	T4a N0 M0
IIC	T4b N0 M0
IIIA	T1-2 N1 M0
	T1 N2a M0
IIIB	T3-4a N1 M0
	T2-3 N2a M0
	T1-2 N2b M0
IIIC	T4a N2a M0
	T3-a N2b M0
	T4b N1-2 M0
IVA	Tx Nx M1a
IVB	Tx Nx M1b
IVC	Tx Nx M1c
